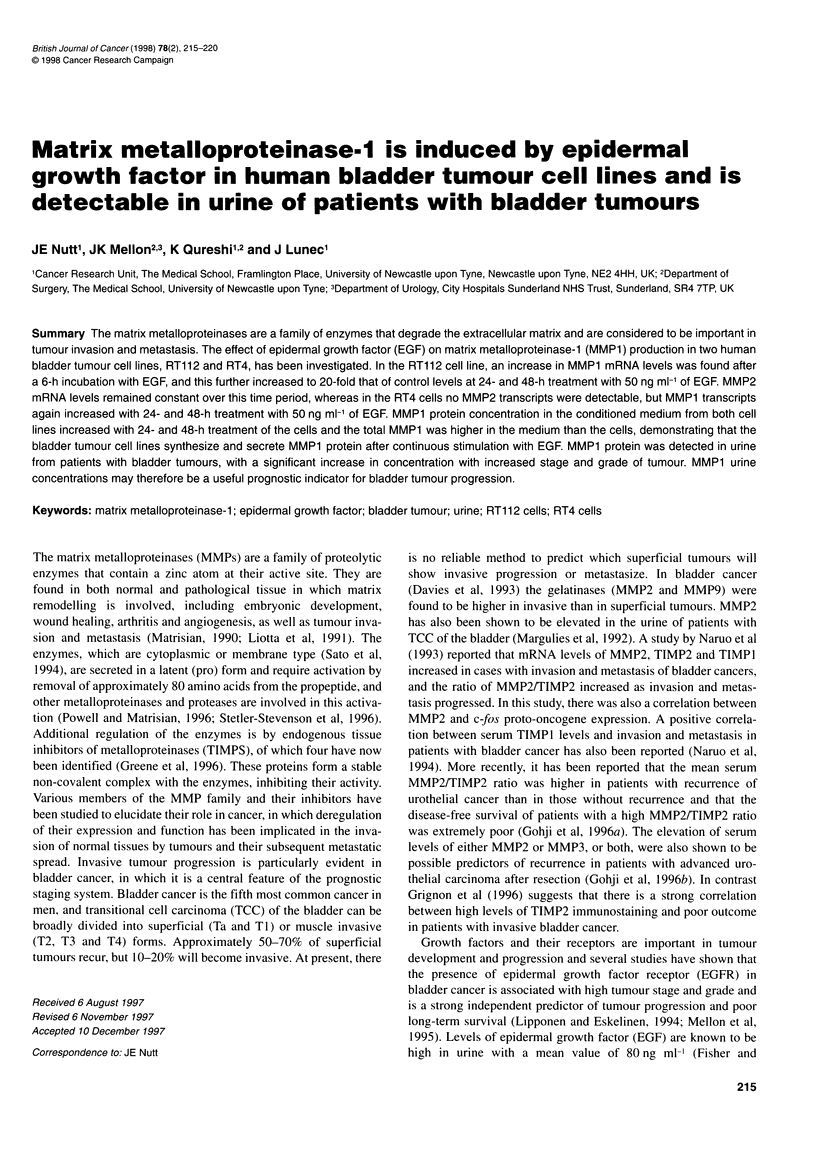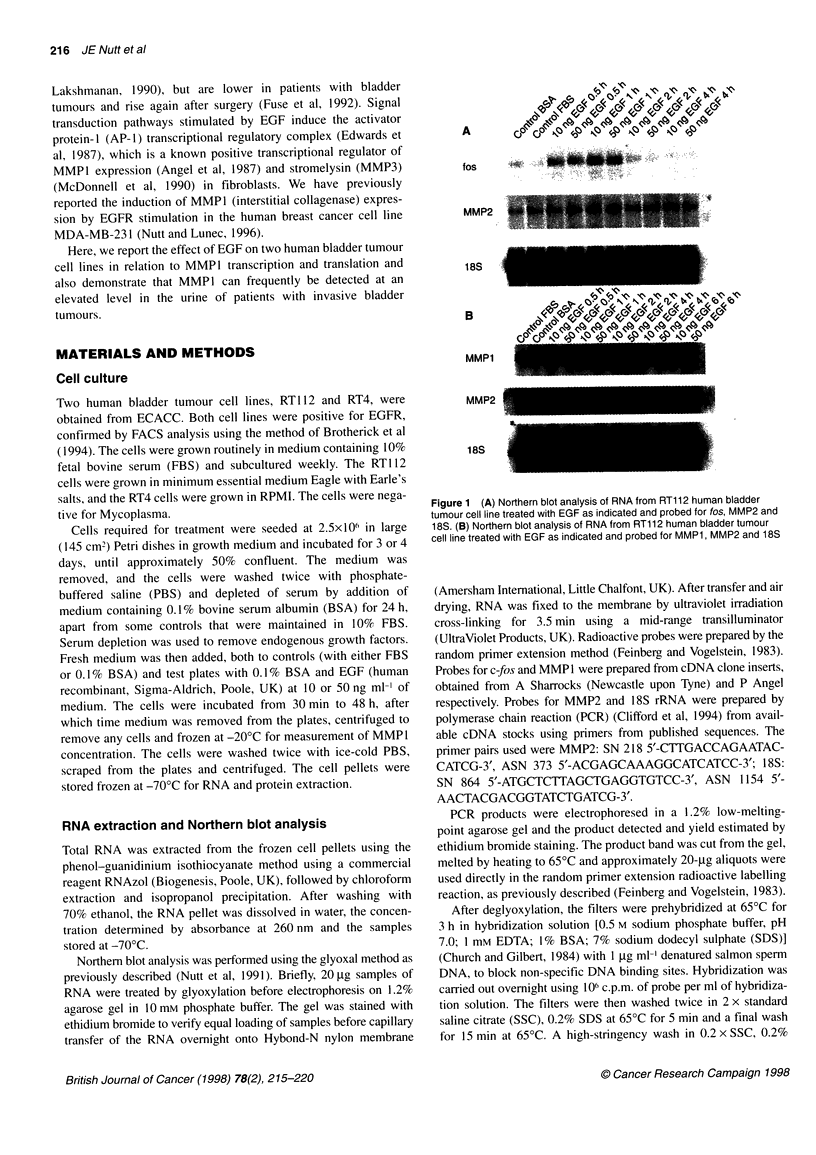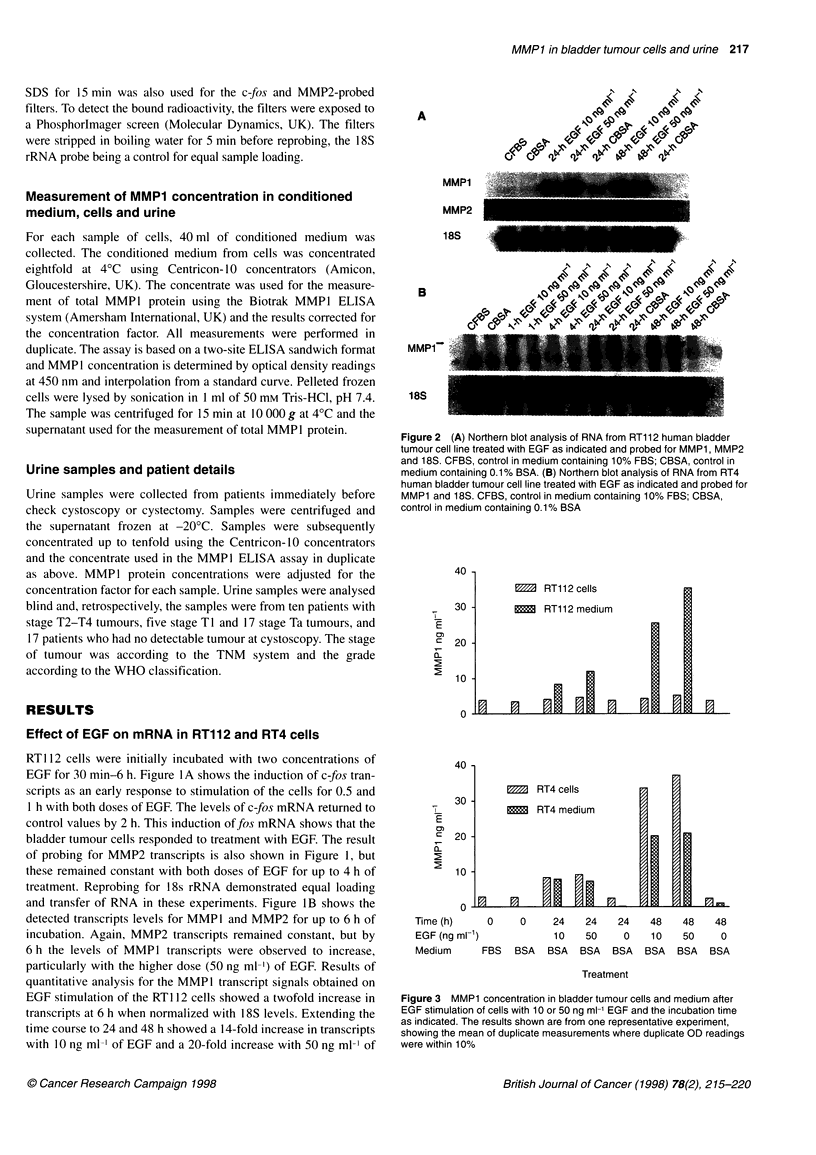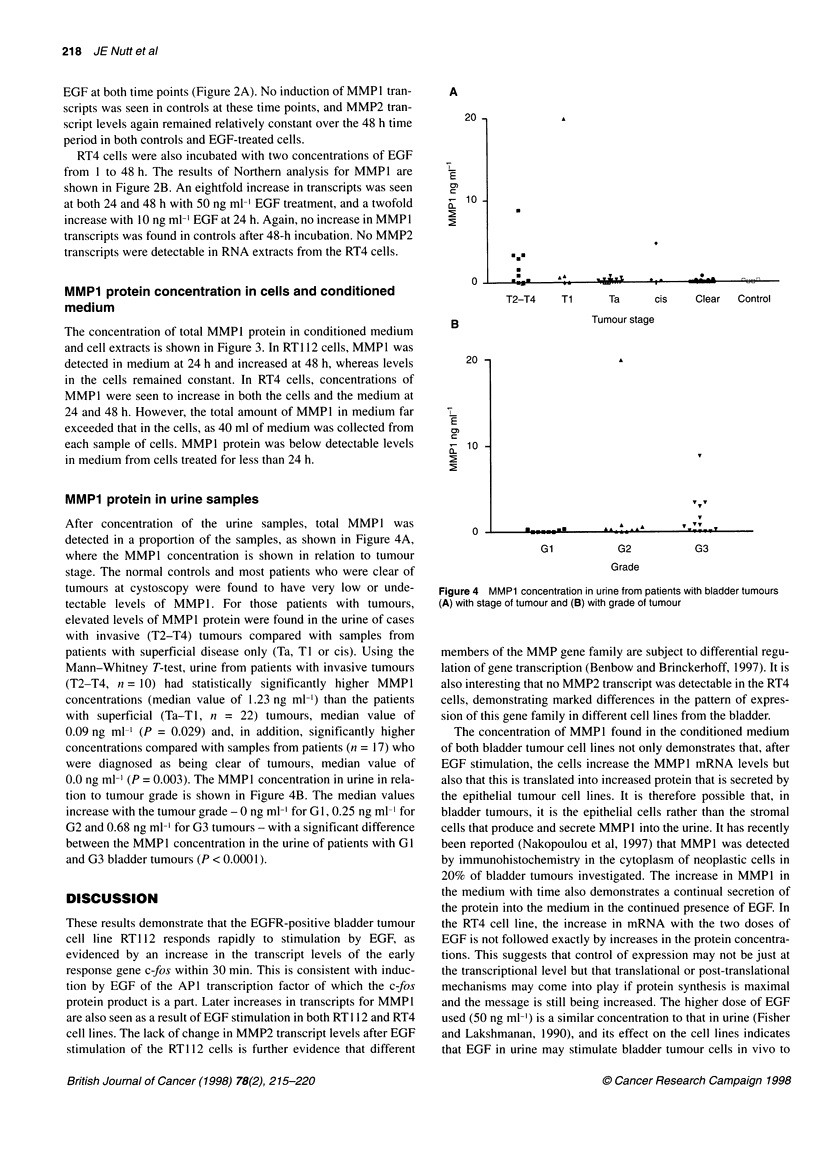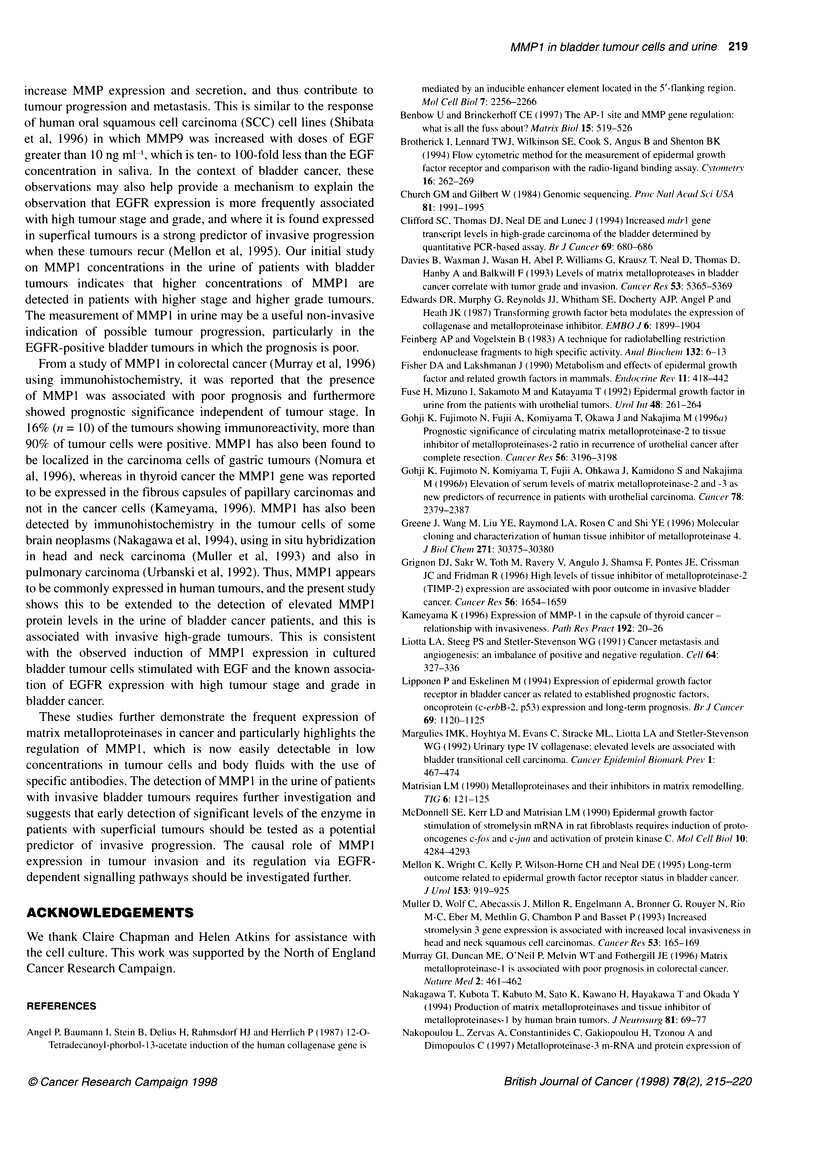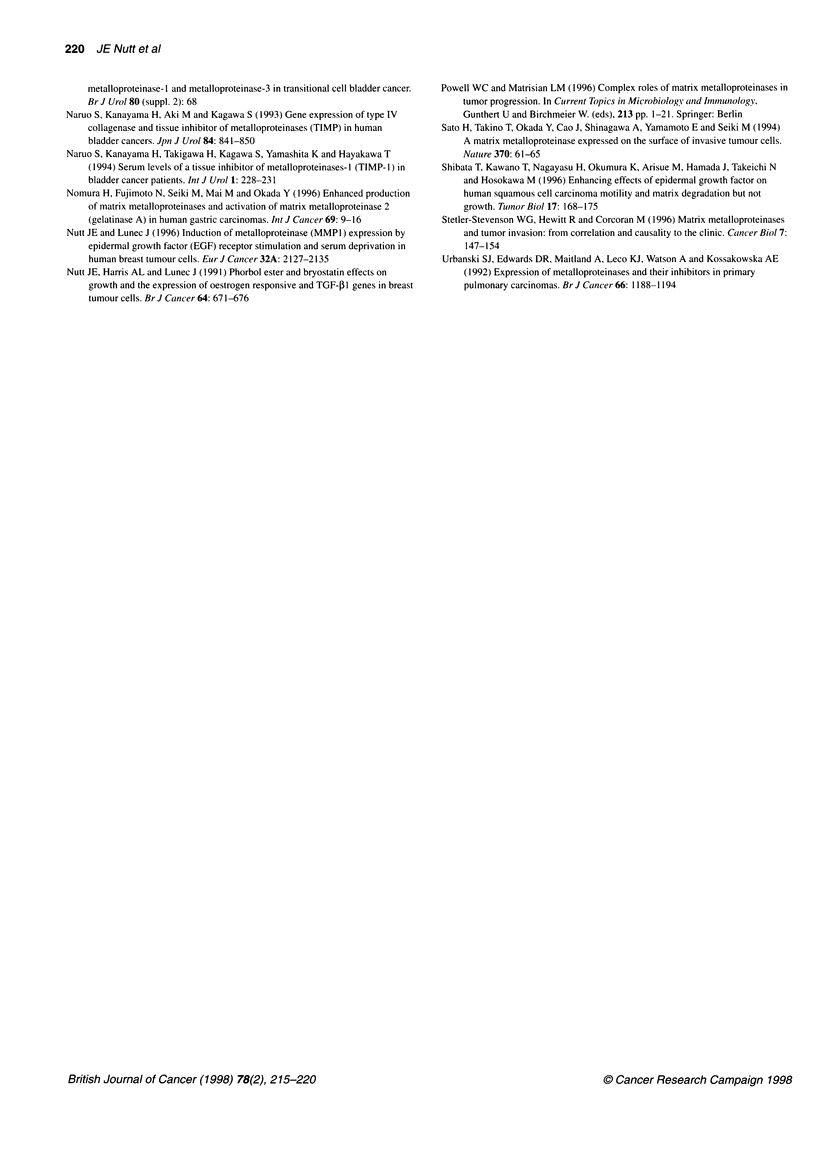# Matrix metalloproteinase-1 is induced by epidermal growth factor in human bladder tumour cell lines and is detectable in urine of patients with bladder tumours.

**DOI:** 10.1038/bjc.1998.467

**Published:** 1998-07

**Authors:** J. E. Nutt, J. K. Mellon, K. Qureshi, J. Lunec

**Affiliations:** Cancer Research Unit, The Medical School, Framlington Place, University of Newcastle upon Tyne, UK.

## Abstract

**Images:**


British Journal of Cancer (1998) 78(2), 215–220
© 1998 Cancer Research Campaign

### Matrix metalloproteinase-1 is induced by epidermal growth factor in human bladder tumour cell lines and is detectable in urine of patients with bladder tumours

**JE Nutt[1], JK Mellon[2,3], K Qureshi[1,2] and J Lunec[1]**

[1]Cancer Research Unit, The Medical School, Framlington Place, University of Newcastle upon Tyne, Newcastle upon Tyne, NE2 4HH, UK; [2]Department of Surgery, The Medical School, University of Newcastle upon Tyne; [3]Department of Urology, City Hospitals Sunderland NHS Trust, Sunderland, SR4 7TP, UK

**Summary** The matrix metalloproteinases are a family of enzymes that degrade the extracellular matrix and are considered to be important in tumour invasion and metastasis. The effect of epidermal growth factor (EGF) on matrix metalloproteinase-1 (MMP1) production in two human bladder tumour cell lines, RT112 and RT4, has been investigated. In the RT112 cell line, an increase in MMP1 mRNA levels was found after a 6-h incubation with EGF, and this further increased to 20-fold that of control levels at 24- and 48-h treatment with 50 ng ml$^{-1}$ of EGF. MMP2 mRNA levels remained constant over this time period, whereas in the RT4 cells no MMP2 transcripts were detectable, but MMP1 transcripts again increased with 24- and 48-h treatment with 50 ng ml$^{-1}$ of EGF. MMP1 protein concentration in the conditioned medium from both cell lines increased with 24- and 48-h treatment of the cells and the total MMP1 was higher in the medium than the cells, demonstrating that the bladder tumour cell lines synthesize and secrete MMP1 protein after continuous stimulation with EGF. MMP1 protein was detected in urine from patients with bladder tumours, with a significant increase in concentration with increased stage and grade of tumour. MMP1 urine concentrations may therefore be a useful prognostic indicator for bladder tumour progression.

**Keywords:** matrix metalloproteinase-1; epidermal growth factor; bladder tumour; urine; RT112 cells; RT4 cells

The matrix metalloproteinases (MMPs) are a family of proteolytic enzymes that contain a zinc atom at their active site. They are found in both normal and pathological tissue in which matrix remodelling is involved, including embryonic development, wound healing, arthritis and angiogenesis, as well as tumour invasion and metastasis (Matrisian, 1990; Liotta et al, 1991). The enzymes, which are cytoplasmic or membrane type (Sato et al, 1994), are secreted in a latent (pro) form and require activation by removal of approximately 80 amino acids from the propeptide, and other metalloproteinases and proteases are involved in this activation (Powell and Matrisian, 1996; Stetler-Stevenson et al, 1996). Additional regulation of the enzymes is by endogenous tissue inhibitors of metalloproteinases (TIMPS), of which four have now been identified (Greene et al, 1996). These proteins form a stable non-covalent complex with the enzymes, inhibiting their activity. Various members of the MMP family and their inhibitors have been studied to elucidate their role in cancer, in which deregulation of their expression and function has been implicated in the invasion of normal tissues by tumours and their subsequent metastatic spread. Invasive tumour progression is particularly evident in bladder cancer, in which it is a central feature of the prognostic staging system. Bladder cancer is the fifth most common cancer in men, and transitional cell carcinoma (TCC) of the bladder can be broadly divided into superficial (Ta and T1) or muscle invasive (T2, T3 and T4) forms. Approximately 50–70% of superficial tumours recur, but 10–20% will become invasive. At present, there is no reliable method to predict which superficial tumours will show invasive progression or metastasize. In bladder cancer (Davies et al, 1993) the gelatinases (MMP2 and MMP9) were found to be higher in invasive than in superficial tumours. MMP2 has also been shown to be elevated in the urine of patients with TCC of the bladder (Margulies et al, 1992). A study by Naruo et al (1993) reported that mRNA levels of MMP2, TIMP2 and TIMP1 increased in cases with invasion and metastasis of bladder cancers, and the ratio of MMP2/TIMP2 increased as invasion and metastasis progressed. In this study, there was also a correlation between MMP2 and c-*fos* proto-oncogene expression. A positive correlation between serum TIMP1 levels and invasion and metastasis in patients with bladder cancer has also been reported (Naruo et al, 1994). More recently, it has been reported that the mean serum MMP2/TIMP2 ratio was higher in patients with recurrence of urothelial cancer than in those without recurrence and that the disease-free survival of patients with a high MMP2/TIMP2 ratio was extremely poor (Gohji et al, 1996*a*). The elevation of serum levels of either MMP2 or MMP3, or both, were also shown to be possible predictors of recurrence in patients with advanced urothelial carcinoma after resection (Gohji et al, 1996*b*). In contrast Grignon et al (1996) suggests that there is a strong correlation between high levels of TIMP2 immunostaining and poor outcome in patients with invasive bladder cancer.

Growth factors and their receptors are important in tumour development and progression and several studies have shown that the presence of epidermal growth factor receptor (EGFR) in bladder cancer is associated with high tumour stage and grade and is a strong independent predictor of tumour progression and poor long-term survival (Lipponen and Eskelinen, 1994; Mellon et al, 1995). Levels of epidermal growth factor (EGF) are known to be high in urine with a mean value of 80 ng ml$^{-1}$ (Fisher and

*Received 6 August 1997*
*Revised 6 November 1997*
*Accepted 10 December 1997*

*Correspondence to:* JE Nutt

Lakshmanan, 1990), but are lower in patients with bladder tumours and rise again after surgery (Fuse et al, 1992). Signal transduction pathways stimulated by EGF induce the activator protein-1 (AP-1) transcriptional regulatory complex (Edwards et al, 1987), which is a known positive transcriptional regulator of MMP1 expression (Angel et al, 1987) and stromelysin (MMP3) (McDonnell et al, 1990) in fibroblasts. We have previously reported the induction of MMP1 (interstitial collagenase) expression by EGFR stimulation in the human breast cancer cell line MDA-MB-231 (Nutt and Lunec, 1996).

Here, we report the effect of EGF on two human bladder tumour cell lines in relation to MMP1 transcription and translation and also demonstrate that MMP1 can frequently be detected at an elevated level in the urine of patients with invasive bladder tumours.

### MATERIALS AND METHODS

### Cell culture

Two human bladder tumour cell lines, RT112 and RT4, were obtained from ECACC. Both cell lines were positive for EGFR, confirmed by FACS analysis using the method of Brotherick et al (1994). The cells were grown routinely in medium containing 10% fetal bovine serum (FBS) and subcultured weekly. The RT112 cells were grown in minimum essential medium Eagle with Earle's salts, and the RT4 cells were grown in RPMI. The cells were negative for Mycoplasma.

Cells required for treatment were seeded at $2.5 \times 10^6$ in large (145 $cm^2$) Petri dishes in growth medium and incubated for 3 or 4 days, until approximately 50% confluent. The medium was removed, and the cells were washed twice with phosphate-buffered saline (PBS) and depleted of serum by addition of medium containing 0.1% bovine serum albumin (BSA) for 24 h, apart from some controls that were maintained in 10% FBS. Serum depletion was used to remove endogenous growth factors. Fresh medium was then added, both to controls (with either FBS or 0.1% BSA) and test plates with 0.1% BSA and EGF (human recombinant, Sigma-Aldrich, Poole, UK) at 10 or 50 ng $ml^{-1}$ of medium. The cells were incubated from 30 min to 48 h, after which time medium was removed from the plates, centrifuged to remove any cells and frozen at −20°C for measurement of MMP1 concentration. The cells were washed twice with ice-cold PBS, scraped from the plates and centrifuged. The cell pellets were stored frozen at −70°C for RNA and protein extraction.

### RNA extraction and Northern blot analysis

Total RNA was extracted from the frozen cell pellets using the phenol–guanidinium isothiocyanate method using a commercial reagent RNAzol (Biogenesis, Poole, UK), followed by chloroform extraction and isopropanol precipitation. After washing with 70% ethanol, the RNA pellet was dissolved in water, the concentration determined by absorbance at 260 nm and the samples stored at −70°C.

Northern blot analysis was performed using the glyoxal method as previously described (Nutt et al, 1991). Briefly, 20 μg samples of RNA were treated by glyoxylation before electrophoresis on 1.2% agarose gel in 10 mM phosphate buffer. The gel was stained with ethidium bromide to verify equal loading of samples before capillary transfer of the RNA overnight onto Hybond-N nylon membrane

**Figure 1** (**A**) Northern blot analysis of RNA from RT112 human bladder tumour cell line treated with EGF as indicated and probed for *fos*, MMP2 and 18S. (**B**) Northern blot analysis of RNA from RT112 human bladder tumour cell line treated with EGF as indicated and probed for MMP1, MMP2 and 18S

(Amersham International, Little Chalfont, UK). After transfer and air drying, RNA was fixed to the membrane by ultraviolet irradiation cross-linking for 3.5 min using a mid-range transilluminator (UltraViolet Products, UK). Radioactive probes were prepared by the random primer extension method (Feinberg and Vogelstein, 1983). Probes for c-*fos* and MMP1 were prepared from cDNA clone inserts, obtained from A Sharrocks (Newcastle upon Tyne) and P Angel respectively. Probes for MMP2 and 18S rRNA were prepared by polymerase chain reaction (PCR) (Clifford et al, 1994) from available cDNA stocks using primers from published sequences. The primer pairs used were MMP2: SN 218 5′-CTTGACCAGAATACCATCG-3′, ASN 373 5′-ACGAGCAAAGGCATCATCC-3′; 18S: SN 864 5′-ATGCTCTTAGCTGAGGTGTCC-3′, ASN 1154 5′-AACTACGACGGTATCTGATCG-3′.

PCR products were electrophoresed in a 1.2% low-melting-point agarose gel and the product detected and yield estimated by ethidium bromide staining. The product band was cut from the gel, melted by heating to 65°C and approximately 20-μg aliquots were used directly in the random primer extension radioactive labelling reaction, as previously described (Feinberg and Vogelstein, 1983).

After deglyoxylation, the filters were prehybridized at 65°C for 3 h in hybridization solution [0.5 M sodium phosphate buffer, pH 7.0; 1 mM EDTA; 1% BSA; 7% sodium dodecyl sulphate (SDS)] (Church and Gilbert, 1984) with 1 μg $ml^{-1}$ denatured salmon sperm DNA, to block non-specific DNA binding sites. Hybridization was carried out overnight using $10^6$ c.p.m. of probe per ml of hybridization solution. The filters were then washed twice in 2 × standard saline citrate (SSC), 0.2% SDS at 65°C for 5 min and a final wash for 15 min at 65°C. A high-stringency wash in 0.2 × SSC, 0.2%

© Cancer Research Campaign 1998

SDS for 15 min was also used for the c-*fos* and MMP2-probed filters. To detect the bound radioactivity, the filters were exposed to a PhosphorImager screen (Molecular Dynamics, UK). The filters were stripped in boiling water for 5 min before reprobing, the 18S rRNA probe being a control for equal sample loading.

### Measurement of MMP1 concentration in conditioned medium, cells and urine

For each sample of cells, 40 ml of conditioned medium was collected. The conditioned medium from cells was concentrated eightfold at 4°C using Centricon-10 concentrators (Amicon, Gloucestershire, UK). The concentrate was used for the measurement of total MMP1 protein using the Biotrak MMP1 ELISA system (Amersham International, UK) and the results corrected for the concentration factor. All measurements were performed in duplicate. The assay is based on a two-site ELISA sandwich format and MMP1 concentration is determined by optical density readings at 450 nm and interpolation from a standard curve. Pelleted frozen cells were lysed by sonication in 1 ml of 50 mM Tris-HCl, pH 7.4. The sample was centrifuged for 15 min at 10 000 ***g*** at 4°C and the supernatant used for the measurement of total MMP1 protein.

### Urine samples and patient details

Urine samples were collected from patients immediately before check cystoscopy or cystectomy. Samples were centrifuged and the supernatant frozen at –20°C. Samples were subsequently concentrated up to tenfold using the Centricon-10 concentrators and the concentrate used in the MMP1 ELISA assay in duplicate as above. MMP1 protein concentrations were adjusted for the concentration factor for each sample. Urine samples were analysed blind and, retrospectively, the samples were from ten patients with stage T2–T4 tumours, five stage T1 and 17 stage Ta tumours, and 17 patients who had no detectable tumour at cystoscopy. The stage of tumour was according to the TNM system and the grade according to the WHO classification.

### RESULTS

### Effect of EGF on mRNA in RT112 and RT4 cells

RT112 cells were initially incubated with two concentrations of EGF for 30 min–6 h. Figure 1A shows the induction of c-*fos* transcripts as an early response to stimulation of the cells for 0.5 and 1 h with both doses of EGF. The levels of c-*fos* mRNA returned to control values by 2 h. This induction of *fos* mRNA shows that the bladder tumour cells responded to treatment with EGF. The result of probing for MMP2 transcripts is also shown in Figure 1, but these remained constant with both doses of EGF for up to 4 h of treatment. Reprobing for 18s rRNA demonstrated equal loading and transfer of RNA in these experiments. Figure 1B shows the detected transcripts levels for MMP1 and MMP2 for up to 6 h of incubation. Again, MMP2 transcripts remained constant, but by 6 h the levels of MMP1 transcripts were observed to increase, particularly with the higher dose (50 ng ml$^{-1}$) of EGF. Results of quantitative analysis for the MMP1 transcript signals obtained on EGF stimulation of the RT112 cells showed a twofold increase in transcripts at 6 h when normalized with 18S levels. Extending the time course to 24 and 48 h showed a 14-fold increase in transcripts with 10 ng ml$^{-1}$ of EGF and a 20-fold increase with 50 ng ml$^{-1}$ of

**Figure 2** (**A**) Northern blot analysis of RNA from RT112 human bladder tumour cell line treated with EGF as indicated and probed for MMP1, MMP2 and 18S. CFBS, control in medium containing 10% FBS; CBSA, control in medium containing 0.1% BSA. (**B**) Northern blot analysis of RNA from RT4 human bladder tumour cell line treated with EGF as indicated and probed for MMP1 and 18S. CFBS, control in medium containing 10% FBS; CBSA, control in medium containing 0.1% BSA

**Figure 3** MMP1 concentration in bladder tumour cells and medium after EGF stimulation of cells with 10 or 50 ng ml$^{-1}$ EGF and the incubation time as indicated. The results shown are from one representative experiment, showing the mean of duplicate measurements where duplicate OD readings were within 10%

© Cancer Research Campaign 1998

EGF at both time points (Figure 2A). No induction of MMP1 transcripts was seen in controls at these time points, and MMP2 transcript levels again remained relatively constant over the 48 h time period in both controls and EGF-treated cells.

RT4 cells were also incubated with two concentrations of EGF from 1 to 48 h. The results of Northern analysis for MMP1 are shown in Figure 2B. An eightfold increase in transcripts was seen at both 24 and 48 h with 50 ng $ml^{-1}$ EGF treatment, and a twofold increase with 10 ng $ml^{-1}$ EGF at 24 h. Again, no increase in MMP1 transcripts was found in controls after 48-h incubation. No MMP2 transcripts were detectable in RNA extracts from the RT4 cells.

### MMP1 protein concentration in cells and conditioned medium

The concentration of total MMP1 protein in conditioned medium and cell extracts is shown in Figure 3. In RT112 cells, MMP1 was detected in medium at 24 h and increased at 48 h, whereas levels in the cells remained constant. In RT4 cells, concentrations of MMP1 were seen to increase in both the cells and the medium at 24 and 48 h. However, the total amount of MMP1 in medium far exceeded that in the cells, as 40 ml of medium was collected from each sample of cells. MMP1 protein was below detectable levels in medium from cells treated for less than 24 h.

### MMP1 protein in urine samples

After concentration of the urine samples, total MMP1 was detected in a proportion of the samples, as shown in Figure 4A, where the MMP1 concentration is shown in relation to tumour stage. The normal controls and most patients who were clear of tumours at cystoscopy were found to have very low or undetectable levels of MMP1. For those patients with tumours, elevated levels of MMP1 protein were found in the urine of cases with invasive (T2–T4) tumours compared with samples from patients with superficial disease only (Ta, T1 or cis). Using the Mann–Whitney *T*-test, urine from patients with invasive tumours (T2–T4, $n = 10$) had statistically significantly higher MMP1 concentrations (median value of 1.23 ng $ml^{-1}$) than the patients with superficial (Ta–T1, $n = 22$) tumours, median value of 0.09 ng $ml^{-1}$ ($P = 0.029$) and, in addition, significantly higher concentrations compared with samples from patients ($n = 17$) who were diagnosed as being clear of tumours, median value of 0.0 ng $ml^{-1}$ ($P = 0.003$). The MMP1 concentration in urine in relation to tumour grade is shown in Figure 4B. The median values increase with the tumour grade – 0 ng $ml^{-1}$ for G1, 0.25 ng $ml^{-1}$ for G2 and 0.68 ng $ml^{-1}$ for G3 tumours – with a significant difference between the MMP1 concentration in the urine of patients with G1 and G3 bladder tumours ($P < 0.0001$).

**Figure 4** MMP1 concentration in urine from patients with bladder tumours (**A**) with stage of tumour and (**B**) with grade of tumour

### DISCUSSION

These results demonstrate that the EGFR-positive bladder tumour cell line RT112 responds rapidly to stimulation by EGF, as evidenced by an increase in the transcript levels of the early response gene c-*fos* within 30 min. This is consistent with induction by EGF of the AP1 transcription factor of which the c-*fos* protein product is a part. Later increases in transcripts for MMP1 are also seen as a result of EGF stimulation in both RT112 and RT4 cell lines. The lack of change in MMP2 transcript levels after EGF stimulation of the RT112 cells is further evidence that different members of the MMP gene family are subject to differential regulation of gene transcription (Benbow and Brinckerhoff, 1997). It is also interesting that no MMP2 transcript was detectable in the RT4 cells, demonstrating marked differences in the pattern of expression of this gene family in different cell lines from the bladder.

The concentration of MMP1 found in the conditioned medium of both bladder tumour cell lines not only demonstrates that, after EGF stimulation, the cells increase the MMP1 mRNA levels but also that this is translated into increased protein that is secreted by the epithelial tumour cell lines. It is therefore possible that, in bladder tumours, it is the epithelial cells rather than the stromal cells that produce and secrete MMP1 into the urine. It has recently been reported (Nakopoulou et al, 1997) that MMP1 was detected by immunohistochemistry in the cytoplasm of neoplastic cells in 20% of bladder tumours investigated. The increase in MMP1 in the medium with time also demonstrates a continual secretion of the protein into the medium in the continued presence of EGF. In the RT4 cell line, the increase in mRNA with the two doses of EGF is not followed exactly by increases in the protein concentrations. This suggests that control of expression may not be just at the transcriptional level but that translational or post-translational mechanisms may come into play if protein synthesis is maximal and the message is still being increased. The higher dose of EGF used (50 ng $ml^{-1}$) is a similar concentration to that in urine (Fisher and Lakshmanan, 1990), and its effect on the cell lines indicates that EGF in urine may stimulate bladder tumour cells in vivo to

 © Cancer Research Campaign 1998

increase MMP expression and secretion, and thus contribute to tumour progression and metastasis. This is similar to the response of human oral squamous cell carcinoma (SCC) cell lines (Shibata et al, 1996) in which MMP9 was increased with doses of EGF greater than 10 ng $ml^{-1}$, which is ten- to 100-fold less than the EGF concentration in saliva. In the context of bladder cancer, these observations may also help provide a mechanism to explain the observation that EGFR expression is more frequently associated with high tumour stage and grade, and where it is found expressed in superfical tumours is a strong predictor of invasive progression when these tumours recur (Mellon et al, 1995). Our initial study on MMP1 concentrations in the urine of patients with bladder tumours indicates that higher concentrations of MMP1 are detected in patients with higher stage and higher grade tumours. The measurement of MMP1 in urine may be a useful non-invasive indication of possible tumour progression, particularly in the EGFR-positive bladder tumours in which the prognosis is poor.

From a study of MMP1 in colorectal cancer (Murray et al, 1996) using immunohistochemistry, it was reported that the presence of MMP1 was associated with poor prognosis and furthermore showed prognostic significance independent of tumour stage. In 16% ($n$ = 10) of the tumours showing immunoreactivity, more than 90% of tumour cells were positive. MMP1 has also been found to be localized in the carcinoma cells of gastric tumours (Nomura et al, 1996), whereas in thyroid cancer the MMP1 gene was reported to be expressed in the fibrous capsules of papillary carcinomas and not in the cancer cells (Kameyama, 1996). MMP1 has also been detected by immunohistochemistry in the tumour cells of some brain neoplasms (Nakagawa et al, 1994), using in situ hybridization in head and neck carcinoma (Muller et al, 1993) and also in pulmonary carcinoma (Urbanski et al, 1992). Thus, MMP1 appears to be commonly expressed in human tumours, and the present study shows this to be extended to the detection of elevated MMP1 protein levels in the urine of bladder cancer patients, and this is associated with invasive high-grade tumours. This is consistent with the observed induction of MMP1 expression in cultured bladder tumour cells stimulated with EGF and the known association of EGFR expression with high tumour stage and grade in bladder cancer.

These studies further demonstrate the frequent expression of matrix metalloproteinases in cancer and particularly highlights the regulation of MMP1, which is now easily detectable in low concentrations in tumour cells and body fluids with the use of specific antibodies. The detection of MMP1 in the urine of patients with invasive bladder tumours requires further investigation and suggests that early detection of significant levels of the enzyme in patients with superficial tumours should be tested as a potential predictor of invasive progression. The causal role of MMP1 expression in tumour invasion and its regulation via EGFR-dependent signalling pathways should be investigated further.

### ACKNOWLEDGEMENTS

We thank Claire Chapman and Helen Atkins for assistance with the cell culture. This work was supported by the North of England Cancer Research Campaign.

### REFERENCES

Angel P, Baumann I, Stein B, Delius H, Rahmsdorf HJ and Herrlich P (1987) 12-O-Tetradecanoyl-phorbol-13-acetate induction of the human collagenase gene is mediated by an inducible enhancer element located in the 5′-flanking region. *Mol Cell Biol* **7**: 2256–2266

Benbow U and Brinckerhoff CE (1997) The AP-1 site and MMP gene regulation: what is all the fuss about? *Matrix Biol* **15**: 519–526

Brotherick I, Lennard TWJ, Wilkinson SE, Cook S, Angus B and Shenton BK (1994) Flow cytometric method for the measurement of epidermal growth factor receptor and comparison with the radio-ligand binding assay. *Cytometry* **16**: 262–269

Church GM and Gilbert W (1984) Genomic sequencing. *Proc Natl Acad Sci USA* **81**: 1991–1995

Clifford SC, Thomas DJ, Neal DE and Lunec J (1994) Increased *mdr*1 gene transcript levels in high-grade carcinoma of the bladder determined by quantitative PCR-based assay. *Br J Cancer* **69**: 680–686

Davies B, Waxman J, Wasan H, Abel P, Williams G, Krausz T, Neal D, Thomas D, Hanby A and Balkwill F (1993) Levels of matrix metalloproteases in bladder cancer correlate with tumor grade and invasion. *Cancer Res* **53**: 5365–5369

Edwards DR, Murphy G, Reynolds JJ, Whitham SE, Docherty AJP, Angel P and Heath JK (1987) Transforming growth factor beta modulates the expression of collagenase and metalloproteinase inhibitor. *EMBO J* **6**: 1899–1904

Feinberg AP and Vogelstein B (1983) A technique for radiolabelling restriction endonuclease fragments to high specific activity. *Anal Biochem* **132**: 6–13

Fisher DA and Lakshmanan J (1990) Metabolism and effects of epidermal growth factor and related growth factors in mammals. *Endocrine Rev* **11**: 418–442

Fuse H, Mizuno I, Sakamoto M and Katayama T (1992) Epidermal growth factor in urine from the patients with urothelial tumors. *Urol Int* **48**: 261–264

Gohji K, Fujimoto N, Fujii A, Komiyama T, Okawa J and Nakajima M (1996*a*) Prognostic significance of circulating matrix metalloproteinase-2 to tissue inhibitor of metalloproteinases-2 ratio in recurrence of urothelial cancer after complete resection. *Cancer Res* **56**: 3196–3198

Gohji K, Fujimoto N, Komiyama T, Fujii A, Ohkawa J, Kamidono S and Nakajima M (1996*b*) Elevation of serum levels of matrix metalloproteinase-2 and -3 as new predictors of recurrence in patients with urothelial carcinoma. *Cancer* **78**: 2379–2387

Greene J, Wang M, Liu YE, Raymond LA, Rosen C and Shi YE (1996) Molecular cloning and characterization of human tissue inhibitor of metalloproteinase 4. *J Biol Chem* **271**: 30375–30380

Grignon DJ, Sakr W, Toth M, Ravery V, Angulo J, Shamsa F, Pontes JE, Crissman JC and Fridman R (1996) High levels of tissue inhibitor of metalloproteinase-2 (TIMP-2) expression are associated with poor outcome in invasive bladder cancer. *Cancer Res* **56**: 1654–1659

Kameyama K (1996) Expression of MMP-1 in the capsule of thyroid cancer – relationship with invasiveness. *Path Res Pract* **192**: 20–26

Liotta LA, Steeg PS and Stetler-Stevenson WG (1991) Cancer metastasis and angiogenesis: an imbalance of positive and negative regulation. *Cell* **64**: 327–336

Lipponen P and Eskelinen M (1994) Expression of epidermal growth factor receptor in bladder cancer as related to established prognostic factors, oncoprotein (c-*erb*B-2, p53) expression and long-term prognosis. *Br J Cancer* **69**: 1120–1125

Margulies IMK, Hoyhtya M, Evans C, Stracke ML, Liotta LA and Stetler-Stevenson WG (1992) Urinary type IV collagenase: elevated levels are associated with bladder transitional cell carcinoma. *Cancer Epidemiol Biomark Prev* **1**: 467–474

Matrisian LM (1990) Metalloproteinases and their inhibitors in matrix remodelling. *TIG* **6**: 121–125

McDonnell SE, Kerr LD and Matrisian LM (1990) Epidermal growth factor stimulation of stromelysin mRNA in rat fibroblasts requires induction of proto-oncogenes c-*fos* and c-*jun* and activation of protein kinase C. *Mol Cell Biol* **10**: 4284–4293

Mellon K, Wright C, Kelly P, Wilson-Horne CH and Neal DE (1995) Long-term outcome related to epidermal growth factor receptor status in bladder cancer. *J Urol* **153**: 919–925

Muller D, Wolf C, Abecassis J, Millon R, Engelmann A, Bronner G, Rouyer N, Rio M-C, Eber M, Methlin G, Chambon P and Basset P (1993) Increased stromelysin 3 gene expression is associated with increased local invasiveness in head and neck squamous cell carcinomas. *Cancer Res* **53**: 165–169

Murray GI, Duncan ME, O'Neil P, Melvin WT and Fothergill JE (1996) Matrix metalloproteinase-1 is associated with poor prognosis in colorectal cancer. *Nature Med* **2**: 461–462

Nakagawa T, Kubota T, Kabuto M, Sato K, Kawano H, Hayakawa T and Okada Y (1994) Production of matrix metalloproteinases and tissue inhibitor of metalloproteinases-1 by human brain tumors. *J Neurosurg* **81**: 69–77

Nakopoulou L, Zervas A, Constantinides C, Gakiopoulou H, Tzonou A and Dimopoulos C (1997) Metalloproteinase-3 m-RNA and protein expression of

© Cancer Research Campaign 1998

metalloproteinase-1 and metalloproteinase-3 in transitional cell bladder cancer. *Br J Urol* **80** (suppl. 2): 68

Naruo S, Kanayama H, Aki M and Kagawa S (1993) Gene expression of type IV collagenase and tissue inhibitor of metalloproteinases (TIMP) in human bladder cancers. *Jpn J Urol* **84**: 841–850

Naruo S, Kanayama H, Takigawa H, Kagawa S, Yamashita K and Hayakawa T (1994) Serum levels of a tissue inhibitor of metalloproteinases-1 (TIMP-1) in bladder cancer patients. *Int J Urol* **1**: 228–231

Nomura H, Fujimoto N, Seiki M, Mai M and Okada Y (1996) Enhanced production of matrix metalloproteinases and activation of matrix metalloproteinase 2 (gelatinase A) in human gastric carcinomas. *Int J Cancer* **69**: 9–16

Nutt JE and Lunec J (1996) Induction of metalloproteinase (MMP1) expression by epidermal growth factor (EGF) receptor stimulation and serum deprivation in human breast tumour cells. *Eur J Cancer* **32A**: 2127–2135

Nutt JE, Harris AL and Lunec J (1991) Phorbol ester and bryostatin effects on growth and the expression of oestrogen responsive and TGF-β1 genes in breast tumour cells. *Br J Cancer* **64**: 671–676

Powell WC and Matrisian LM (1996) Complex roles of matrix metalloproteinases in tumor progression. In *Current Topics in Microbiology and Immunology*, Gunthert U and Birchmeier W. (eds), **213** pp. 1–21. Springer: Berlin

Sato H, Takino T, Okada Y, Cao J, Shinagawa A, Yamamoto E and Seiki M (1994) A matrix metalloproteinase expressed on the surface of invasive tumour cells. *Nature* **370**: 61–65

Shibata T, Kawano T, Nagayasu H, Okumura K, Arisue M, Hamada J, Takeichi N and Hosokawa M (1996) Enhancing effects of epidermal growth factor on human squamous cell carcinoma motility and matrix degradation but not growth. *Tumor Biol* **17**: 168–175

Stetler-Stevenson WG, Hewitt R and Corcoran M (1996) Matrix metalloproteinases and tumor invasion: from correlation and causality to the clinic. *Cancer Biol* **7**: 147–154

Urbanski SJ, Edwards DR, Maitland A, Leco KJ, Watson A and Kossakowska AE (1992) Expression of metalloproteinases and their inhibitors in primary pulmonary carcinomas. *Br J Cancer* **66**: 1188–1194

© Cancer Research Campaign 1998